# The Central Role of *KNG1* Gene as a Genetic Determinant of Coagulation Pathway-Related Traits: Exploring *Metaphenotypes*

**DOI:** 10.1371/journal.pone.0167187

**Published:** 2016-12-22

**Authors:** Helena Brunel, Raimon Massanet, Angel Martinez-Perez, Andrey Ziyatdinov, Laura Martin-Fernandez, Juan Carlos Souto, Alexandre Perera, José Manuel Soria

**Affiliations:** 1 Unit of Genomics of Complex Diseases, Sant Pau Institute of Biomedical Research (IIB-Sant Pau), Barcelona, Spain; 2 B2SLab, Departament d’Enginyeria de Sistemes, Automàtica i Informàtica Industrial, Universitat Politècnica de Catalunya (UPC), Barcelona, Spain; 3 Thrombosis and Haemostasis Unit, Sant Pau Institute of Biomedical Research (IIB-Sant Pau), Barcelona, Spain; University of Texas MD Anderson Cancer Center, UNITED STATES

## Abstract

Traditional genetic studies of single traits may be unable to detect the pleiotropic effects involved in complex diseases. To detect the correlation that exists between several phenotypes involved in the same biological process, we introduce an original methodology to analyze sets of correlated phenotypes involved in the coagulation cascade in genome-wide association studies. The methodology consists of a two-stage process. First, we define new phenotypic meta-variables (linear combinations of the original phenotypes), named *metaphenotypes*, by applying Independent Component Analysis for the multivariate analysis of correlated phenotypes (i.e. the levels of coagulation pathway–related proteins). The resulting *metaphenotypes* integrate the information regarding the underlying biological process (i.e. thrombus/clot formation). Secondly, we take advantage of a family based Genome Wide Association Study to identify genetic elements influencing these *metaphenotypes* and consequently thrombosis risk. Our study utilized data from the GAIT Project (Genetic Analysis of Idiopathic Thrombophilia). We obtained 15 *metaphenotypes*, which showed significant heritabilities, ranging from 0.2 to 0.7. These results indicate the importance of genetic factors in the variability of these traits. We found 4 *metaphenotypes* that showed significant associations with SNPs. The most relevant were those mapped in a region near the *HRG*, *FETUB* and *KNG1* genes. Our results are provocative since they show that the *KNG1* locus plays a central role as a genetic determinant of the entire coagulation pathway and thrombus/clot formation. Integrating data from multiple correlated measurements through *metaphenotypes* is a promising approach to elucidate the hidden genetic mechanisms underlying complex diseases.

## Introduction

Considerable efforts have been invested to evaluate hundreds of genetic variants associated with human traits. Despite these efforts, the loci that have been identified only explain a small proportion of the total phenotypic variance. Thus, there is the question of where the remaining heritability resides. For a complex disease, such as thrombosis, traditional single-trait genetic studies may be unable to detect the pleiotropic effect that a given genetic variant could have on the intermediate phenotypes involved with the disease. In particular, the normal physiological process underlying thrombosis is complex and many of its components are involved in the coagulation and fibrinolysis pathways. These components form a collection of intermediate phenotypes that are generally measured in the study of thrombosis. These intermediate phenotypes may reflect more directly the effects from causal genes than disease status. They are also less genetically complex and more strongly associated with susceptibility loci.

So far, the genetic analyses of thrombosis have been carried out using one or more intermediate traits separately [1–7]. However, if a locus is associated with two or more traits, i.e. it is pleiotropic, a single-trait study may lose the power to detect this pleiotropic effect. However, finding disease risk indexes would contribute to a greater understanding of the pathogenesis of disease, and ultimately will develop better diagnostic, prevention and treatment strategies. In addition, the simultaneous analyses of multiple traits may uncover regulating elements such as master regulators or variants belonging to transcription factor binding sites. Genetic analyses have been performed using aPTT (Activated Partial Thromboplastin Time) as a phenotype to improve the understanding of the biological mechanisms underlying thrombotic disease [8,9]. Although aPTT measures the combined activity of several clotting factors in the intrinsic and common coagulation pathways [10] (including factors FII, FV, FVIII, FIX, FX, FXI and FXII), the present genetic studies on aPTT consider it as an univariate model without considering pleiotropic effects [11]. Another example of exploiting the genetic information of different traits comes from the GAIT (Genetic Analysis of Idiopathic Thrombophilia) Project, where we demonstrated that coagulation factors FVIII and vWF are genetically correlated with thrombotic disease [12]. Also, in a previous study, we identified common variants associated with the plasma levels of several proteins and consequently the risk of thrombosis [13]. However, the pleiotropic effects of loci in the coagulation cascade have not been explored fully.

Both genetic association and linkage research have focused on statistical and computational techniques to investigate the genetic effects between one genotype and one phenotype including polygenic and multiphenotypic approaches. Several strategies have been applied for the analysis of multiple and correlated traits. These can be divided into three categories: p-value correction methods, regression models and data reduction methods. P-value correction methods consist on combining several univariate tests, one for each trait, accounting for the observed correlational structure of the traits [14,15].

Regression models make use of mixed effects models for modelling the covariance structure of the phenotypes, as well as population structure [16]. These two approaches have a limited practical use since with a large number of correlated traits, they require the simultaneous estimation of too many parameters [17]. As an alternative, data reduction methods based on the transformation of the original traits to a reduced number of canonical traits have been proposed [18–20] with the intent of applying the traditional single trait analyses to these new variables. Generally, the canonical variables are obtained through a given mathematical model that transforms the original phenotypic data in a new space of reduced dimensionality where the new coordinate axes (also called components) define new phenotypic quantities obtained synthetically. In particular, Principal Components Analysis (PCA) has been applied for this purpose [17, 21, 22].

In this study, we explore an original methodology to determine the inner correlation within a set of related traits involved in the coagulation cascade, to help understanding the genetic bases of the coagulation cascade consequently of thrombosis risk. We apply Independent Component Analysis, a data reduction method, original in this field, to derive new phenotypic variables, called *metaphenotypes*, which integrate information regarding the underlying biological variability on the thrombus/clot formation. Then, we take advantage of our GWAS to identify genetic elements influencing these *metaphenotypes* and their relationship with thrombosis risk.

## Materials and Methods

### The GAIT Project

The GAIT (Genetic Analysis of Idiopathic Thrombophilia) Project has been described in Souto et al 2000 [13]. Briefly, the GAIT Project included 398 individuals from 21 extended Spanish families (mean pedigree size = 19) [12]. Twelve of these families were selected on the basis of a proband with idiopathic thrombophilia, whereas the remaining nine families were unaffected and selected randomly. The ages of the subjects ranged from <1 to 88 years (mean = 37.7 years) and the male to female sex ratio was 0.85. The study was performed according to the Declaration of Helsinki. All procedures were reviewed by the Institutional Review Board of the Hospital de la Santa Creu i Sant Pau, Barcelona, Spain. Adult subjects gave written consent for themselves and for their minor children.

#### Genotypes and Data Cleaning

A genome-wide set of 307,984 SNPs was typed for all of the participants using the Infinium^®^ 317k Beadchip on the Illumina platform (San Diego, CA, USA). Individuals with a low call rate (<0.5%), a too high IBS (>0.95%) and a too high heterozygosity (FDR <1%) were removed from the sample. In addition, markers with a low call rate (<0.95%) and a low MAF (<0.0064%) were discarded also. A total of 34 individuals and 30,793 SNPs were removed from the study. A clean dataset containing *n* = 364 individuals and 277,191 SNPs was obtained for further analyses. This procedure was implemented in R using the GenABEL package [23].

#### Phenotypes

Among the 80 phenotypes in the GAIT sample, *m* = 27 phenotypes involved in the coagulation pathway were selected to study their joint biological activity within this metabolic process. These phenotypes were selected as they are defined in the literature [24]. The original phenotypes are described in S1 Table.

To properly apply the mathematical methods that we used, phenotypic data were freed of missing values. To guarantee this condition, the phenotypic dataset was imputed using a bPCA, a Bayesian method for missing value imputation [25].

### “Metaphenotypes” as a Concept

A *metaphenotype* is defined as a new phenotypic variable obtained synthetically from a set of traits (phenotypes) using a given mathematical model of dimensionality reduction. *Metaphenotypes* should be able to capture the original structure of the data to describe them as a whole. Therefore, identifying genetic variants related to these *metaphenotypes* may help to ascertain the genetic bases of the observed variability of the set of phenotypes, here the coagulation pathway.

The coagulation factors in the coagulation cascade show related patterns of activity. It is known that the genes coding for the different coagulation factors share a joint ancestry [13], so there may exist also some regulatory elements jointly regulating their activity. We consider analyzing the 27 coagulation pathway-related phenotypes measured in the GAIT project under the concept of *metaphenotypes*.

*Metaphenotypes* are computed from the correlation among factors. There are several algorithms in the literature that are able to decompose the variability under different criteria. We applied an ICA (Independent Component Analysis), an algorithm based on a criterion of minimum shared information. ICA was compared to PCA(Principal Component Analysis) a reference method for studying the genetic association of correlated phenotypes [18, 21].

### Statistical Analyses

Both PCA and ICA methods apply a linear transformation to the original phenotypic data and obtain a new system of coordinates of reduced dimensionality, following the expression in Eq 1.

X=M ⋅ W+E (1)

where X (n × m) are the original phenotypes, M (n × m) are the *metaphenotypes* and W (m × m) are the weights of the model and E is the error of the model. Note that the *metaphenotypes* correspond to the axes of the new system of coordinates, and are called “components”. The maximum number of components obtained is the same as the original phenotypes but generally, only a few of them are informative and therefore are taken into account.

The *metaphenotypes* are determined by the characterization of the weights of this linear transformation, either using PCA or using ICA.

#### Independent Component Analysis

In ICA, the weights W are optimized to guarantee the statistical independence of the *metaphenotypes*. The independence of the components is guaranteed by finding W that maximizes the non-gaussianity of the *metaphenotypes* (M).

Among the several ICA algorithms, the fastICA procedure was applied, using a particular approximation of the negentropy measure for maximizing the nongaussianity [26]. In particular, this method was applied with an optimal number of *metaphenotypes* (components) of *k = 15*, according to a criterion based on cross-validation approximations [27]. As other ICA implementations, fastICA previously applies a PCA to the data in order to ensure that the components are uncorrelated. The number of components to be used are then determined from the PCA procedure using a cross-validation model.

#### Principal Component Analysis

In PCA, the weights W are optimized so that the *metaphenotypes* capture the maximum covariance existing between the original phenotypes. In this case, the *metaphenotypes* explore the correlation that exists among the original traits to capture the variability shared by the collection of original phenotypes.

PCA was used as a reference method. By default, PCA obtained as many components as original variables (k = 27).

#### Differences between PCA and ICA

As the structure of the interrelations among phenotypes is hidden and unknown, both techniques are complementary to unravel the cascade of physiological relationships.

PCA and ICA answer different biological questions.

PCA obtains *metaphenotypes* that explain the greatest overall variability or correlation between the original phenotypes. In other words, *metaphenotypes* built with PCA are a new set of indexes of jointly altered levels of the original phenotypes, capturing the common activity of the original phenotypes.

In contrast, ICA was chosen because it obtains *metaphenotypes* that are statistically independent. Thus, ICA is able to separate the different (independent) sources of variability captured by the original phenotypes. Let us consider the original traits as statistical mixtures of different sources of variability (genetic, environmental, or experimental). If there was a genetic source of variability captured by the set of phenotypes (pleiotropy), the *metaphenotypes* obtained using ICA will capture it. In other words, ICA is especially useful to detect pleiotropic effects.

#### Heritability Estimation

The heritabilities of the *metaphenotypes* were estimated using the variance component method implemented in SOLAR [28]. This method partitions the total phenotypic variance into a proportion due to polygenic (additive) effects and a proportion due to environmental effects. The heritability (*h2r*) estimates the total variance of a trait due to additive genetic effects.

#### Genetic Association

Genome-Wide Association Analyses with the SNPs of the GAIT project were performed using a Likelihood Ratio test based on a linear mixed effects (Variance Components) model described in Eq 2.

The model provides a vector of fitted values of the phenotype and an estimate of the variance-covariance matrix for each family [28, 29, 30].

The polygenic mixed model defined in Eq 2 was applied for each *metaphenotype* M_i_ with the age and gender co-variables for testing the association as they present a significant correlation with almost all of the *metaphenotypes*.

$$M_{i}∼\mu +\Sigma_{j}\beta_{j}c_{ji}+G_{i}+\varepsilon_{j}$$ (2)

where i is the individual index, M_i_ is the *metaphenotype*, μ is the overall mean, β_j_ is the regression coefficient of the j-th covariate, c_ji_ is the j-th covariate, G_i_ is the random additive polygenic effect (breeding value) which variance is defined as Φσ_G_ where Φ is the kinship matrix and σ_G_ is the additive genetic variance due to polygenes. Finally ε_i_ are the residuals of the model.

With a comparative purpose, GWAS of the original phenotypes were also computed.

All the p-values where corrected using the Bonferroni critertion, with a significance criterion set at α = 0.05 after adjustment.

## Results

A total of 15 *metaphenotypes* were obtained with our methodology. All of the *metaphenotypes* showed a significant heritability ranging from 0.15 to 0.7 (Table 1). Significant findings obtained in GWAS are shown in Table 2. To illustrate the relevance of these findings, they were compared with metaphenotypes obtained with a PCA-based approach and with univariate GWAS applied to the original phenotypes. Heritabilities of PCA-based metaphenotypes are shown in S2 Table. Table 2 presents SNPs significantly associated with ICA-based metaphenotypes in comparison with PCA-based *metaphenotypes* and univariate phenotypes. For two particular SNPs (*rs9898* and *rs27311672*), concordant results were found among the three GWAS approaches. Both SNPs were significantly associated with both an ICA-based and a PCA-based *metaphenotype* as well as with the univariate phenotypes corresponding to the proteins coded by their respective closest gene (*HRG* and *F12*). Concordant ICA-based and PCA-based metaphenotypes were compared as follows. Associations p-values with all the original phenotypes with the SNPs reported in Table 2 are presented in S3 Table.

**Table 1:** Heritabilities of ICA-based metaphenotypes (components 1 to 15 from the ICA model).

Metaphenotype	h2r
C1	0.48***
C2	0.17*
C3	0.53***
C4	0.15*
C5	0.22*
C6	0.61***
C7	0.24**
C8	0.55***
C9	0.35***
C10	0.7***
C11	0.45***
C12	0.58***
C13	0.32***
C14	0.24***
C15	0.59***

Significant thresholds for heritability estimation:

* <0.05,

**<0.005,

*** <0.0005

**Table 2:** GWAS significant SNPs for the three approaches (univariate phenotypes, ICA-based metaphenotypes and PCA-based metaphenotypes. For each SNP, the Chromosome where it is located, its physically closest gene and its MAF are shown as well as the adjusted p-value.

SNP ID	Chr	Gene	MAF	HRG	FXII	P-value						
						ICA—C3	ICA—C4	ICA—C5	*ICA—C10*	PCA—C8	PCA—C9	PCA—C10
rs9898	3	HRG	0.35	1.9 x 10^−16^		9 x 10^−18^				1 x 10^−07^	4.3 x 10^−08^	
rs3733159	3	FETUB	0.34	3.3 x 10^−13^		6.6 x 10^−09^						
rs1621816	3	KNG1	0.24	1.5 x 10^−09^		5 x 10^−08^						
rs1403694	3	KNG1	0.32	1.1 x 10^−08^		6.7 x 10^−07^						
rs17255413	3	BOC	0.007				2.6 x 10^−08^					
rs3113727	4	COL25A1	0.24					3.8 x 10^−07^				
rs27311672	5	F12	0.17		7.6 x 10^−36^				1.1 x 10^−14^			1.5 x 10^−11^

**Fig 1:**
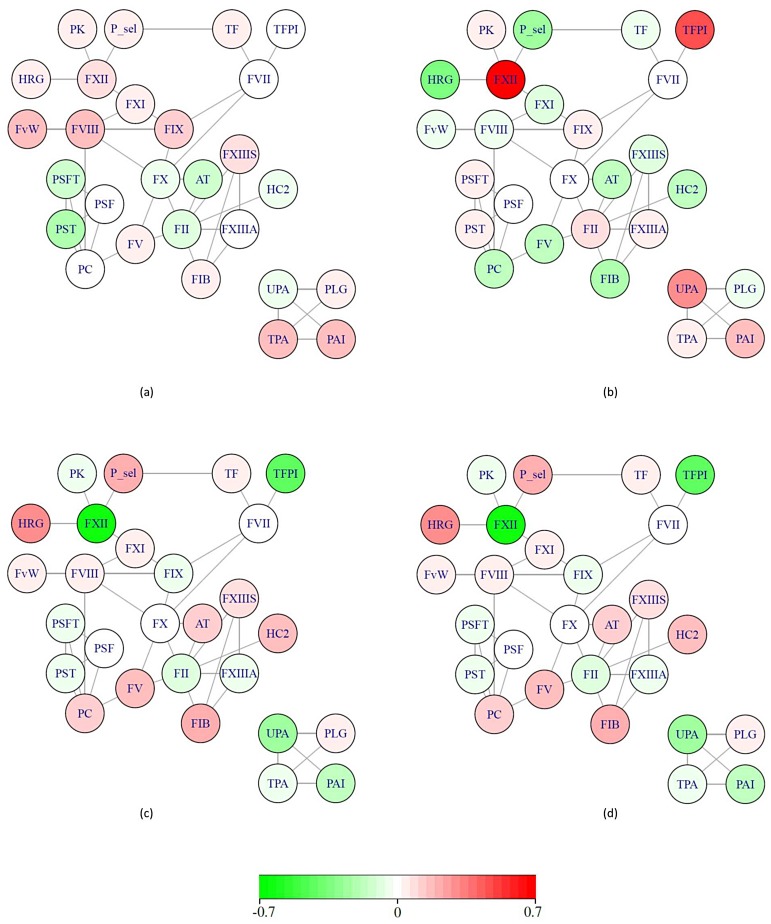
Metaphenotype graphical representation using a simple graph. (a) ICA-based metaphenotype corresponding to the 3^rd^ component (ICA-C3), (b) ICA-C10, (c) PCA-C9 (d) PCA-C10.

To obtain a clear and interpretable view of *metaphenotypes*, we plotted them in a simple graph (Fig 1). Non-directed graphs were used to express the existing interaction among the 27 original phenotypes, represented by nodes whose colors represent their weights in the resulting *metaphenotype*. This is interpretable as the contribution of the original phenotype to the corresponding *metaphenotype*. Numerical values for the weights are included in S4 and S5 Tables.

It is observed in Table 2 that ICA and PCA obtained concordant in two cases. For instance, SNPs *rs2731672* and *rs9898* were significantly associated with metaphenotypes coming from different methodologies.

It is observed that the two metaphenotypes significantly associated with SNP *rs2731672* (ICA-C10 and PCA-C10) are influenced clearly by the trait corresponding to the FXII levels (dark nodes in Fig 1.b and 1.d). This SNP is an intergenic variant ~5.8kb upstream of the *F12* gene. In both cases the FXII levels have an important loading in the metaphenotypes indicating the variability captured by the *metaphenotype* is due highly to the variability in the FXII levels. As expected, this SNP was also significantly associated with the FXII levels with the univariate GWAS approach.

The *metaphenotypes* ICA-C3 and PCA-C9, significantly associated with SNP *rs9898* are shown in Fig 1.a and 1.c. SNP *rs9898* is a nonsynonymous SNP in exon 5 of the *HRG* gene. While the PCA-based *metaphenotype* is oriented clearly to the HRG trait due to the weight of HRG levels in the *metaphenotype* (dark red HRG node in Fig 1.c), this specific trait does not present a high weighting value in the ICA-based *metaphenotype* (Fig 1.a).

**Fig 2:**
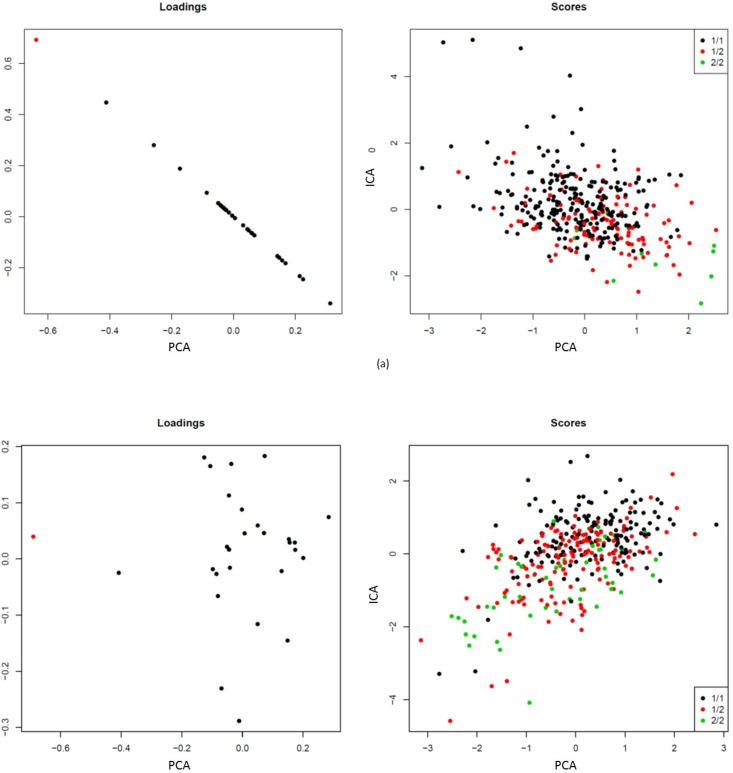
Comparison between the metaphenotypes obtained with both PCA and ICA models. (a) metaphenotype associated with the SNP *rs2731672* at the *F12* locus and (b) metaphenotypes associated with the SNP *rs9898* at the *HRG* locus.

In addition, Fig 2 compares directly both *metaphenotypes* in terms of their loadings (the weight of each trait on the *metaphenotypes*) and their scorings (the projection of each individual on the *metaphenotypes*). For the metaphenotypes associated with SNP *rs2731672* (ICA-C10 and PCA-C10) (Fig 2.a), a clear correlation between both loadings and scorings from both *metaphenotypes* was observed. This confirms that the common variability captured by both *metaphenotypes* is the same in both cases and is due highly to the variability of the FXII. By contrast, as shown in Fig 2.b, no correlation was observed between the loadings or the scoring of the metaphenotypes associated with SNP *rs9898* (ICA-C3 and PCA-C9). This indicates that both metaphenotypes capture different information from the original phenotypes.

In addition, the ICA-C3 metaphenotype was significantly associated with three other SNPs (*rs3733159*, *rs1621816* and *rs1403694*) on Chromosome 3. The former one corresponds to an intronic SNP in the *FETUB* gene, whereas the latter two are intronic SNPs in the *KNG1* gene. It is important to note that *KNG1* is located at a distance of around 40Kb from *HRG*. However, the SNPs *rs1621816* and *rs1403694* in the *KNG1* gene showed a low amount of Linkage Disequilibrium with the SNP *rs9898* in the *HRG* gene (r^2^ = 0.22 and r^2^ = 0.21). These four SNPs showed a significant association with the HRG trait with the univariate GWAS approach.

For the metaphenotypes associated with SNP *rs2731672* (Fig 2.a), a clear correlation between both loadings and scorings from both *metaphenotypes* was observed. This confirms that the common variability captured by both *metaphenotypes* is the same in both cases and is due highly to the variability of the FXII. By contrast, as shown in Fig 2.b, no correlation was observed between the loadings or the scoring indicating that both metaphenotypes capture different information from the original phenotypes.

## Discussion

To date, there are no genetic studies of the coagulation pathway as a whole. Since single-trait genetic studies explain only a small proportion of the phenotypic variability of thrombotic disease, it is prudent to explore other sources of heritability, such as pleiotropy. In our study, we propose a methodology to capture the correlation that exists between a set of intermediate phenotypes involved in the coagulation cascade to elucidate the hidden genetic causes of thrombosis. For doing that, we introduced the concept of *metaphenotype* consisting on new phenotypic indices that gather the observed variability of a collection of related phenotypes. *Metaphenotypes* are obtained through mathematical models of data dimensionality reduction. In this study, we applied ICA for the *metaphenotype* construction. This method is original in this field and was compared to PCA, a reference method for the combined analysis of correlated traits in genetic linkage and association studies^18, 19, 22^. ICA was chosen because it is especially useful to detect pleiotropy. By contrast, PCA is characterized by being able to capture the common variability existing among the phenotypes. Because they answer different biological questions, both methodologies may be complementary.

*Metaphenotypes* were obtained from a collection of coagulation-related phenotypes from the GAIT project^12^ with the aim of identifying genetic variants underlying the whole biological process of blood coagulation. The final goal was to propose genetic markers as candidate regulators of the coagulation cascade and consequently of thrombosis risk.

Even if *metaphenotypes* are not intuitively informative, biologically speaking, they may enable the identification of possibly important loci for a more integrated coagulation index and thus be able to characterize the genetic baseline of coagulation function or thrombosis risk. *Metaphenotypes* can be graphically represented by use of simple graphs (Fig 1) where the original phenotypes involved in their construction are represented by nodes whose colors represent their weights in the resulting *metaphenotype*. This is interpretable as the contribution of the original phenotypes to the corresponding *metaphenotype*. In addition, obtaining metaphenotypes with a heritability greater than 50% (Table 1), allows to assume that the variability of these new phenotypic entities may be highly due to genetic variants. This justifies performing GWAS to *metaphenotypes*.

Results from GWAS with both ICA-based and PCA-based metaphenotypes were concordant in two cases. For instance, SNPs *rs2731672* and *rs9898* were significantly associated with metaphenotypes coming from different methodologies.

In both cases, we compared graphically the *metaphenotype* obtained with ICA and the one obtained with PCA (Fig 2).

As shown in Table 2, the SNP *rs2731672* in the *F12* locus on Chromosome 5 was significantly associated with an ICA-based *metaphenotype* (p-value of 1.1e^-14^) and with a PCA-based *metaphenotype* (p-value: 1.48x10^-11^). It is observed in Fig 1 that both the PCA-based *metaphenotype* (Fig 1.b) and the ICA-based *metaphenotype* (Fig 1.d) are influenced clearly by the FXII levels in blood. In addition, Fig 2.a shows a clear correlation between both loadings and scorings of both *metaphenotypes*. This suggests that the common variability captured by both *metaphenotypes* is the same in both cases and is due highly to the variability of the FXII. This observation is in agreement with the univariate association between this particular *locus* (encoding the structural *F12* gene) and FXII levels [1]. This result confirms that the ICA method also captures non-pleiotropic effects.

Secondly, SNP *rs9898* at the *HRG* locus at chromosome 3 was significantly associated with an ICA-based (p-value: 9e^-18^) and two PCA-based (pvalues: 1e^-07^ and 4.3e^-08^) *metaphenotypes*. Comparisons were carried out with the metaphenotype showing a lower p-value. In this case, no correlation was observed between the loadings or the scoring (Fig 2.b). This suggests that both *metaphenotypes* capture different information of the original traits. Thus, the biological interpretation of the results may be done separately. The univariate GWAS confirmed that SNP *rs9898* is associated with Histidine Rich Glycoprotein (HRG) levels, but previous results also reported that it was associated with Activated Prothrombin Time (aPTT) trait and consequently with thrombosis risk [8, 31]. This explains why, as observed in Fig 1.c, the *metaphenotype* obtained with PCA is oriented clearly to the HRG trait. However, the HRG levels do not have a high weighting value in the *metaphenotype* obtained using ICA (Fig 1.a). In other words, whereas the *metaphenotype* obtained with PCA captures the variance due to the more weighted trait (that is HRG), the result obtained through ICA extend our knowledge about the implication of this genetic variant, indicating that this locus has a pleiotropic effect on the set of coagulation-related traits involved with this *metaphenotype*.

In addition, the same ICA-based *metaphenotype*, showed a significant association with three other SNPs located in the same genomic region of *HRG* on chromosome 3 (rs3733159 at the *FETUB* locus and *rs1621816* and *rs1403694* at the *KNG1* locus). These 3 SNPs were also associated with HRG levels in univariate analyses. The proteins coded by these three genes are Histidine Rich Glycoprotein (HRG), Fetuin-B (FB) and the High Molecular Weight Kininogen (HMWK). All of these proteins are structurally related to a fourth protein, the fetuin A- Heremams Schmide-glycoprotein [32]. Together, they form a subgroup (denoted type 3) within the cystatin superfamily of cysteine inhibitors. Among the several physiological roles associated to type 3 cystatins, the most relevant is the regulation of coagulation and platelet functions, controlled mainly by HRG and kininogen proteins. Furthermore, High-molecular-weight kininogen (HMWK) (encoded by *KNG1*), as well as coagulation Factor FXII (encoded by F12) are, together with prekallikrein (PK), important constituents of the plasma contact-kinin system. This system was first recognized as a surface-activated coagulation system, also known as the Coagulation Intrinsic Pathway (CIP). CIP is activated when blood or plasma interacts with artificial surfaces. A better understanding of this system may lead to insight into mechanisms for thrombosis and, therefore, the contact-kinin system represents a promising multifunctional target for potential thromboembolic therapies, since blocking of distinct members of the kallikrein-kinin system has the potential to become an effective and safe strategy to combat cardiovascular diseases such as myocardial infarction.

Focusing on SNPs located at the *KNG1* locus we observed that SNPs *rs1621816* and *rs1403694* showed a low degree of linkage disequilibrium with the SNP *rs9898* at the *HRG* locus (r^2^ = 0.22 and r^2^ = 0.21). This indicates that they represent an distinct genetic signal. Thus, it is reasonable to suggest that among the results obtained, the association between SNPs located at the *KNG1* locus and an ICA-based *metaphenotype* may have more relevant biological and clinical implications. Our results indicate that KNG1 plays a relevant role in the CIP not only at a molecular level, but also at a genetic level. This result is particularly interesting since allelic variants in *KNG1* were previously associated with risk of thrombosis [33]. Our result strengthens previous conclusions concerning the association of *KNG1* with thrombosis suggesting that *KNG1* plays a role in the regulation of CIP, even without the influence of the FXI or the FXII levels, since neither FXI nor FXII levels show a specific weight within this *metaphenotype* (Fig 1.c).

In conclusion, the methodology proposed in this study complemented existing tools for detecting genetic associations in correlated phenotypes. This strategy explores the potential mechanisms and pathways underlying complex diseases and helps to interpret how they are associated with genetic variants. Our approach is based on the assumption that pleiotropy may occur in many complex diseases and more particularly in thrombosis diseases. The proposed mathematical approach is especially addressed to capture several aspects of the correlated activity of a set of original traits, here blood levels of the proteins involved in the coagulation cascade. Applying this original concept helped to identify two candidate SNPs in the *KNG1* gene susceptible to have an important role in the genetic regulation of the coagulation pathway as a whole and consequently of thrombosis disease.

## Supporting Information

S1 Table Description of the phenotypes. (XLSX)

S2 Table Heritabilities ofPCA-based metaphenotypes (components 1 to 27 from the PCA model). Significant thresholds for heritability estimation: * <0.05, **<0.005, *** <0.0005.
(XLSX)

S3 Table P-values of associations of the relevant SNPs of this study with all the original phenotypes. (XLSX)

S4 Table Loadings (weights) of the coagulation phenotypes in the obtained ICA-based metaphenotypes. (XLSX)

S5 Table Loadings (weights) of the coagulation phenotypes in the obtained PCA-based metaphenotypes. (XLSX)

## References

[1] Soria JM, Almasy L, Souto JC, Bacq D, Buil A, Faure A et al A quantitative-trait locus in the human factor XII gene influences both plasma factor XII levels and susceptibility to thrombotic disease. Am. J. Hum. Genet. 2002;70(3):567–74. 11805911 10.1086/339259 PMC384936

[2] Soria JM, Almasy L, Souto JC, Sabater-Lleal M, Fontcuberta J, Blangero J. The F7 Gene and Clotting Factor VII Levels: Dissection of a Human Quantitative Trait Locus. Hum. Biol. 2009;81(5):853–867. 20504202 10.3378/027.081.0627

[3] Souto JC, Almasy L, Soria JM, Buil A Stone W, Lathrop M et al Genome-wide linkage analysis of von Willebrand factor plasma levels: results from the GAIT Project. Thromb. Haemost. 2003;89(3):468–74. 12624629

[4] Athanasiadis G, Buil A, Souto JC, Borrell M, López S, Martinez-Perez A et al A genome-wide association study of the Protein C anticoagulant pathway. PloS One. 2011;6(12):e29168 10.1371/journal.pone.0029168 22216198 PMC3247258

[5] Soria JM, Almasy L, Souto JC, Buil A, Lathrop M, Blangero J et al A genome search for genetic determinants that influence plasma fibrinogen levels. Arterioscler. Thromb. Vasc. Biol. 2005;25(6):1287–92. 10.1161/01.ATV.0000161927.38739.6f 15761192

[6] Viel KR, Machiah DK, Warren DM, Khachidze M, Buil A, Fernstrom K et al A sequence variation scan of the coagulation factor VIII (FVIII) structural gene and associations with plasma FVIII activity levels. Blood. 2007;109(9):3713–24. 17209060 10.1182/blood-2006-06-026104 PMC1874571

[7] Khachidze M, Buil A, Viel KR, Porter S, Warren D, Machiah DK et al Genetic determinants of normal variation in coagulation factor (F) IX levels: genome-wide scan and examination of the FIX structural gene. J. Thromb. Haemost. 2006;4(7):1537–45. 16839351 10.1111/j.1538-7836.2006.02024.x

[8] Park KJ, Kwon EH, Ma Y, Park IA, Kim SW, Kim SH et al Significantly different coagulation factor activities underlying the variability of “normal” activated partial thromboplastin time. Blood Coagul. Fibrinolysis. 2012;23(1):35–8. 22027757 10.1097/MBC.0b013e32834a6136

[9] Tang W, Schwienbacher C, Lopez LM, Ben-Shlomo Y, Oudot-Mellakh T, Johnson AD et al Genetic associations for activated partial thromboplastin time and prothrombin time, their gene expression profiles, and risk of coronary artery disease. Am. J. Hum. Genet. 2012;91(1):152–62. 22703881 10.1016/j.ajhg.2012.05.009 PMC3397273

[10] Houlihan LM, Davies G, Tenesa A, Harris SE, Luciano M, Gow AJ et al Common variants of large effect in F12, KNG1, and HRG are associated with activated partial thromboplastin time. Am. J. Hum. Genet. 2010;86(4):626–31. 20303064 10.1016/j.ajhg.2010.02.016 PMC2850435

[11] Tang W, Schwienbacher C, Lopez LM, Ben-Shlomo Y, Oudot-Mellakh T, Johnson AD et al, Genetic Associations for Activated Partial Thromboplastin Time and Prothrombin Time, their Gene Expression Profiles, and Risk of Coronary Artery Disease. Am J Hum Genet. 2012 7 13; 91(1): 152–162. 22703881 10.1016/j.ajhg.2012.05.009 PMC3397273

[12] Souto JC, Almasy L, Borrell M, Blanco-Vaca F, Mateo J, Soria JM et al Genetic susceptibility to thrombosis and its relationship to physiological risk factors: the GAIT Study. Am. J. Hum. Genet. 2000;67:1452–9. 11038326 10.1086/316903 PMC1287922

[13] Souto JC, Almasy L, Blangero J, Stone W, Borrell M, Urrutia T et al, Genetic regulation of plasma levels of vitamin K-dependent proteins involved in hematostatis: results from the GAIT Project. Genetic Analysis of Idiopathic Thrombophilia. Thromb Haemost. 2001 1;85(1):88–92. 11204594

[14] Yang Q, Wu H, Guo CY, Fox CS. Analyze multivariate phenotypes in genetic association studies by combining univariate association tests. Genetic Epidemiology. 2010 34:444–54. 10.1002/gepi.20497 20583287 PMC3090041

[15] Xu X, Tian L, Wei LJ. Combining dependent tests for linkage or association across multiple phenotypic traits. Biostatistics. 2003;4(2):223–9. 10.1093/biostatistics/4.2.223 12925518

[16] Stephens M. A unified framework for association analysis with multiple related phenotypes. PLoS ONE. 2013 8:e65245 10.1371/journal.pone.0065245 23861737 PMC3702528

[17] Knott SA, Haley CS. Multitrait Least Squares for Quantitative Trait Loci Detection. Genetics. 2000 156(2):899–911. 11014835 10.1093/genetics/156.2.899 PMC1461263

[18] Klei L, Luca D, Devlin B, Roeder K. Pleiotropy and principal component of heritability combine to increase power for association analysis. Genetic Epidemiology. 2010 34:444–54. 17922480 10.1002/gepi.20257

[19] Mei H, Chen W, Dellinger A, He J, Wang M, Yau C et al Principal-component-based multivariate regression for genetic association studies of metabolic syndrome components. BMC Genet. 2010 11(1):100. 21062472 10.1186/1471-2156-11-100 PMC2991276

[20] Weller JI, Wiggans GR, VanRaden PM, Ron M. Application of a canonical transformation to detection of quantitative trait loci with the aid of genetic markers in a multi-trait experiment. Theor. Appl. Genet. 1996 92(8):998–1002. 10.1007/BF00224040 24166627

[21] Aschard H, Vilhjalmsson BJ, Greliche N, Morange PE, Tregouet DA, Kraft P. Maximizing the power of principal-component analysis of correlated phenotypes in genome wide association studies. American Journal of Human Genetics. 2014 94:662–76. 10.1016/j.ajhg.2014.03.016 24746957 PMC4067564

[22] Mathias RA, Kim Y, Sung H, Yanek LR, Mantese VJ, Hererra-Galeano JE et al A combined genome-wide linkage and association approach to find susceptibility loci for platelet function phenotypes in European American and African American families with coronary artery disease. BMC Med. Genomics. 2010 3:22 10.1186/1755-8794-3-22 20529293 PMC2890666

[23] Aulchenko YS, Ripke S, Isaacs A, van Duijn CM. GenABEL: an R library for genome-wide association analysis. Bioinformatics. 2007 23(10):1294–6. 10.1093/bioinformatics/btm108 17384015

[24] Lefkowitz Jerry B. "Coagulation pathway and physiology" An Algorithmic Approach to Hemostasis Testing. Northfield, IL: College of American Pathologists 2008;3–12.

[25] Stacklies W, Redestig H, Scholz M, Walther D, Selbig J. pcaMethods—a bioconductor package providing PCA methods for incomplete data. Bioinformatics. 2007 23(9):1164–1167. 10.1093/bioinformatics/btm069 17344241

[26] Hyvärinen A, Oja E. Independent component analysis: algorithms and applications. Neural Netw. 2000;(4–5):411–30. 10946390 10.1016/s0893-6080(00)00026-5

[27] Josse J, Husson F. Selecting the number of components in principal component analysis using cross-validation approximations. Comput. Stat. Data Anal. 2012;56(6):1869–1879.

[28] Almasy L, Blangero J. Multipoint quantitative-trait linkage analysis in general pedigrees. Am. J. Hum. Genet. 1998;62(5):1198–211. 10.1086/301844 9545414 PMC1377101

[29] Aulchenko Yurii S., de Koning Dirk-Jan, and Haley Chris. Genomewide rapid association using mixed model and regression: a fast and simple method for genomewide pedigree-based quantitative trait loci association analysis. Genetics. 2007;177(1): 577–585. 10.1534/genetics.107.075614 17660554 PMC2013682

[30] Ziyatdinov A, Brunel H, Martinez-Perez A, Buil A, Perera A, Soria JM. solarius: an R interface to SOLAR for variance component analysis in pedigrees. Bioinformatics. 2016. 10.1093/bioinformatics/btw080 27153684

[31] Morange PE, Oudot-Mellakh T, Cohen W, et al KNG1 Ile581Thr and susceptibility to venous thrombosis. Blood. 2001;117(13): 3692–3694. 10.1182/blood-2010-11-319053 21270443

[32] Lee C., Bongcam-Rudloff E., Sollner C., Jahnen-Dechent W. and Claesson-Welsh L. Type 3 cystatins; fetuins, kininogen and histidine-rich glycoprotein. Frontiers in Bioscience. 2009;14:2911–2922. 10.2741/3422 19273244

[33] Sabater-Lleal M, Martinez-Perez A, Buil A, Folkersen L, Souto JC, Bruzelius M et al A genome-wide association study identifies KNG1 as a genetic determinant of plasma factor XI level and activated Partial Thromboplastin Time. Arterioscler. Thromb. Vasc. Biol. 2012;32:2008–2016. 10.1161/ATVBAHA.112.248492 22701019

